# A hepatoprotective role of peritumoral non-parenchymal cells in early liver tumorigenesis

**DOI:** 10.1242/dmm.049750

**Published:** 2023-03-07

**Authors:** Cheng Tian, Liyuan Li, Li Fan, Anthony Brown, Eric J. Norris, Michelle Morrison, Evan S. Glazer, Liqin Zhu

**Affiliations:** ^1^ Department of Pharmacy and Pharmaceutical Sciences, St. Jude Children's Research Hospital, Memphis, TN 38105, USA; ^2^ STEMCELL Technologies, Vancouver, BC V6A 1B6, Canada; ^3^ Department of Surgery and Cancer Center, College of Medicine, The University of Tennessee Health Science Center, Memphis, TN 38163, USA

**Keywords:** Non-parenchymal cell, Hepatocyte, Spheroid co-culture, Hepatic stellate cell, Liver cancer

## Abstract

Various 3D models of hepatocytes (HCs) have been established to assess liver functions *in vitro*. The contribution of the hepatic non-parenchymal cells (NPCs), however, is largely neglected in these models. Here, we report a comparative study of hepatic spheroids generated from freshly isolated mouse whole liver cells (WLCs) and HCs (referred to as Sph^WLC^ and Sph^HC^, respectively). We found that HC differentiation was preserved better in Sph^WLC^ than in Sph^HC^, and, when co-cultured with liver tumor spheroids (Sph^T^), Sph^WLC^ showed more potent suppression of Sph^T^ growth compared to Sph^HC^. Histological characterization revealed marked activation and accumulation of hepatic stellate cells (HSCs) at the Sph^WLC^:Sph^T^ interface. We found that mixing HSCs in both 3D and 2D HC:tumor co-cultures provided potent protection to HCs against tumor-induced cell death. Activation of HSCs at the tumor border was similarly found in liver tumors from both mice and patients. Overall, our study suggests a hepatoprotective role of peritumoral HSCs in liver tumorigenesis and the potential application of Sph^WLC^ as a useful 3D model for dissecting the liver's response to tumorigenesis *in vitro*.

## Teaser

**Editor's choice:** Our work using *in vitro* liver spheroid models consisting of both parenchymal and non-parenchymal cells shows a hepatoprotective role of peritumoral hepatic stellate cells in liver tumorigenesis.

## INTRODUCTION

The liver is the largest internal organ of the body, with vital functions in metabolism, detoxication, immunity and many more processes. Approximately 80% of the liver mass is composed of parenchymal hepatocytes (HCs), and hepatic non-parenchymal cells (NPCs) – including endothelial cells, Kupffer cells (liver-specific mononuclear phagocyte cells), cholangiocytes, hepatic stellate cells (HSCs; liver-specific fibroblastic cells) and various immune populations (Gao et al., 2008) – represent the remaining 20%. In the normal liver, HCs are responsible for most metabolic and hormonal processes, while NPCs support the structure of the liver, transport molecules in and out, and communicate with the immune system (Malik et al., 2002). The active interplay between the two compartments is known to be critical to normal liver development, damage response and tumorigenesis (Malik et al., 2002; Seo and Jeong, 2016; Myojin et al., 2021; Mohs et al., 2020; Sica et al., 2014). However, this complex, interconnected cell–cell interaction has posed great challenges to our understanding of the individual and coordinated contributions of various hepatic lineages to the development of context-specific liver biology and resultant pathology. There is a need to establish faithful, robust, inclusive and manipulable *in vitro* models that take both HC and NPC compartments into consideration in order to dissect cell–cell interaction in liver biology and pathology.

Recent developments in three-dimensional (3D) *in vitro* cellular models have greatly facilitated research efforts to model and understand normal and malignant development, given their ability to closely mimic the 3D cellular organization of the actual tissue and retain relevant physiology (Tanner and Gottesman, 2015). Organoid and spheroid models are the most commonly used 3D models among various 3D platforms developed, likely because of their easy setup and high efficiency (Chatzinikolaidou, 2016; Driehuis et al., 2020; Sato et al., 2011; Vlachogiannis et al., 2018; Tuveson and Clevers, 2019). For HCs, organoid models are not particularly robust, likely due to the lack of a designated stem cell population supporting HC regeneration (Hu et al., 2018). On the other hand, HC spheroid (Sph^HC^) models are easy to establish and have been widely used as a platform to assess liver function *in vitro* (Mizoi et al., 2020). Recent studies found that chemically defined serum-free medium similar to that used for organoid cultures can maintain the long-term phenotypical and biological stability of Sph^HC^ (Bell et al., 2016; Vorrink et al., 2018; Bao et al., 2013). For NPCs, they have been reported to increase the organization and drug resistance of Sph^HC^ (Baze et al., 2018; Bell et al., 2020). However, studies on spheroids composed of both HC and NPC lineages are limited, representing a missed opportunity to use such spheroid models to understand their differential and coordinated involvement in liver homeostasis and pathogenesis including tumorigenesis.

It has been increasingly recognized that liver cancer is an ecosystem in which tumor cells cooperate with host cells in their intratumoral and peritumoral microenvironment (TME). Although the intratumoral TME of liver cancer, i.e. the stromal components within a liver tumor mass, has been heavily investigated in recent years (Kurebayashi et al., 2021; Job et al., 2020), contribution of its peritumoral TME, or the response of the tumor-adjacent liver tissue to tumorigenesis, is less known. Studies have found widespread cellular and molecular changes in the peritumoral TME of many aggressive types of solid tumors (Xiong et al., 2019; Madden et al., 2020; Ji et al., 2015; D'Alessio et al., 2019; Jiang et al., 2017; Moya et al., 2019; Lee et al., 2018). Interpreting these complex and systemic changes *in vivo* requires appropriate *in vitro* model systems to dissect their individual and coordinated responses to tumorigenesis. Given the presence of many genetically defined mouse models of liver cancer (Newell et al., 2008; Loeuillard et al., 2019), we reasoned that establishing mouse hepatic spheroid models for both normal and malignant liver cells would be beneficial to investigating tumor–liver crosstalk. Thus, in this study, we tested spheroid cultures of unfractionated whole liver cells (WLCs), HCs and NPCs from *B6.129(Cg)-Gt(ROSA)26Sor^tm4(ACTB-tdTomato,-EGFP)Luo^/J* (mTmG) mice and liver tumor spheroids using cells derived from a *Prom1^CreERT2^; Pten^flx/flx^; Tp53^flx/flx^; Rosa-ZsGreen* (PPTR) liver cancer mouse model we previously reported (Zhu et al., 2016; Li et al., 2018). All cells in mTmG mice expressed an intensive tdTomato (tdT) red fluorescence protein, and PPTR tumor cells expressed a strong ZsGreen (ZsG) green fluorescence protein. PPTR tumor cells generated tumors similar to combined hepatocellular carcinoma (HCC) and intrahepatic cholangiocarcinoma (iCCA) upon orthotopic transplantation (Li et al., 2018). HCC and iCCA are the two major liver cancer subtypes that, in rare cases, can be found combined in some patients (Stavraka et al., 2019). A chemically defined serum-free medium was used to grow the spheroids, which were then put in co-cultures to study the response of HCs and NPCs to tumor cells. We confirmed our findings *in vivo* using the PPTR allograft model as well as iCCA and HCC patient tumors.

## RESULTS

### Generation and characterization of mouse primary WLC, HC and NPC spheroids

**Fig. 1.**
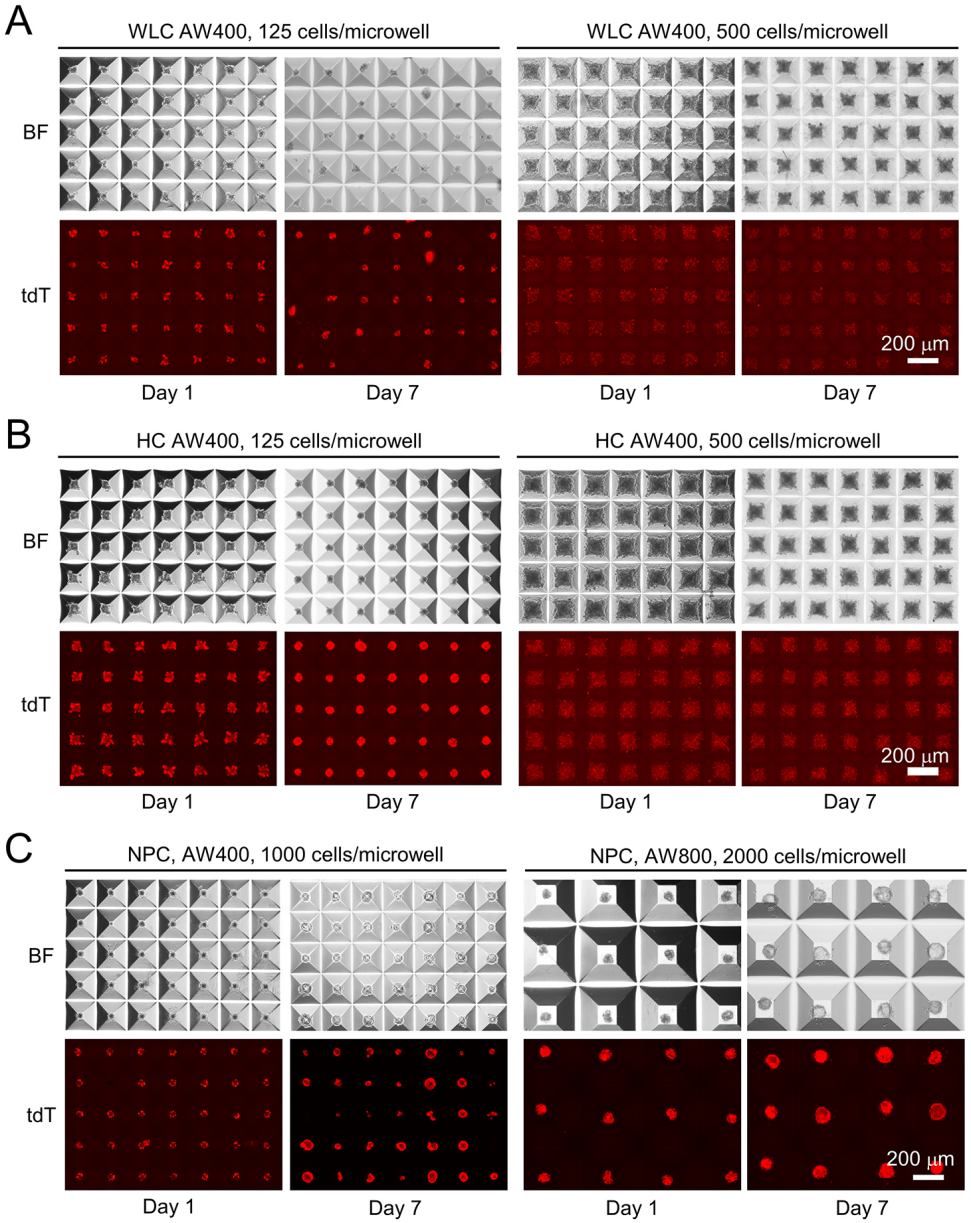
**Generation of whole liver cell (WLC), hepatocyte (HC) and non-parenchymal cell (NPC) spheroids in micropatterned AggreWell (AW) plates.** (A) Day 1 and Day 7 images of freshly isolated tdT^+^ mouse WLCs in AW400 plates seeded at the indicated density. BF, brightfield; tdT, tdTomato fluorescence. (B) Day 1 and Day 7 images of freshly isolated tdT^+^ mouse HCs in AW400 plates seeded at the indicated density. (C) Day 1 and Day 7 images of freshly isolated tdT^+^ mouse NPCs in AW400 and AW800 plates seeded at the indicated density. Spheroid generation was independently validated with liver cells isolated from three mTmG mice. Scale bars: 200 µm.

Single-cell suspension of WLCs was obtained from mTmG mice by standard two-step *in vivo* digestion (Berry and Friend, 1969). HCs and NPCs were then segregated from WLCs by low-speed centrifugation (Fig. S1). Generation of tdT^+^ WLC, HC and NPC spheroids (referred to as Sph^WLC^, Sph^HC^ and Sph^NPC^, respectively) was optimized by seeding in AggreWell 400 (AW400) microplates at 125, 250, 500 and 1000 cells/microwell and AggreWell 800 (AW800) microplates at 500, 1000 and 2000 cells/microwell. A 1:1 mixture of HepatiCult organoid culture medium and a reported cholangiocyte organoid culture medium was used in all cultures to maintain an inclusive and consistent spheroid culture condition. We found that WLCs and HCs behaved similarly and formed spheroids most consistently at 125 cells/microwell in AW400 plates after 2-3 days and were maintained up to 7 days in this study (Fig. 1A,B). Seeding densities ≥500 cells/microwell prevented HC and WLC aggregation as spheroids (Fig. 1A,B). NPCs at 1000 cells/microwell in AW400 plates and 2000 cells/microwell in AW800 plates showed consistent spheroid formation in 5-7 days, and we used the latter condition for all follow-up assays (Fig. 1C). NPCs aggregated poorly at densities lower than 1000 cells/microwell, likely due to their smaller sizes compared with HCs. Different from the solid spheroids formed by HCs and WLCs, NPC spheroids were mostly hollow in structure.

**Fig. 2.**
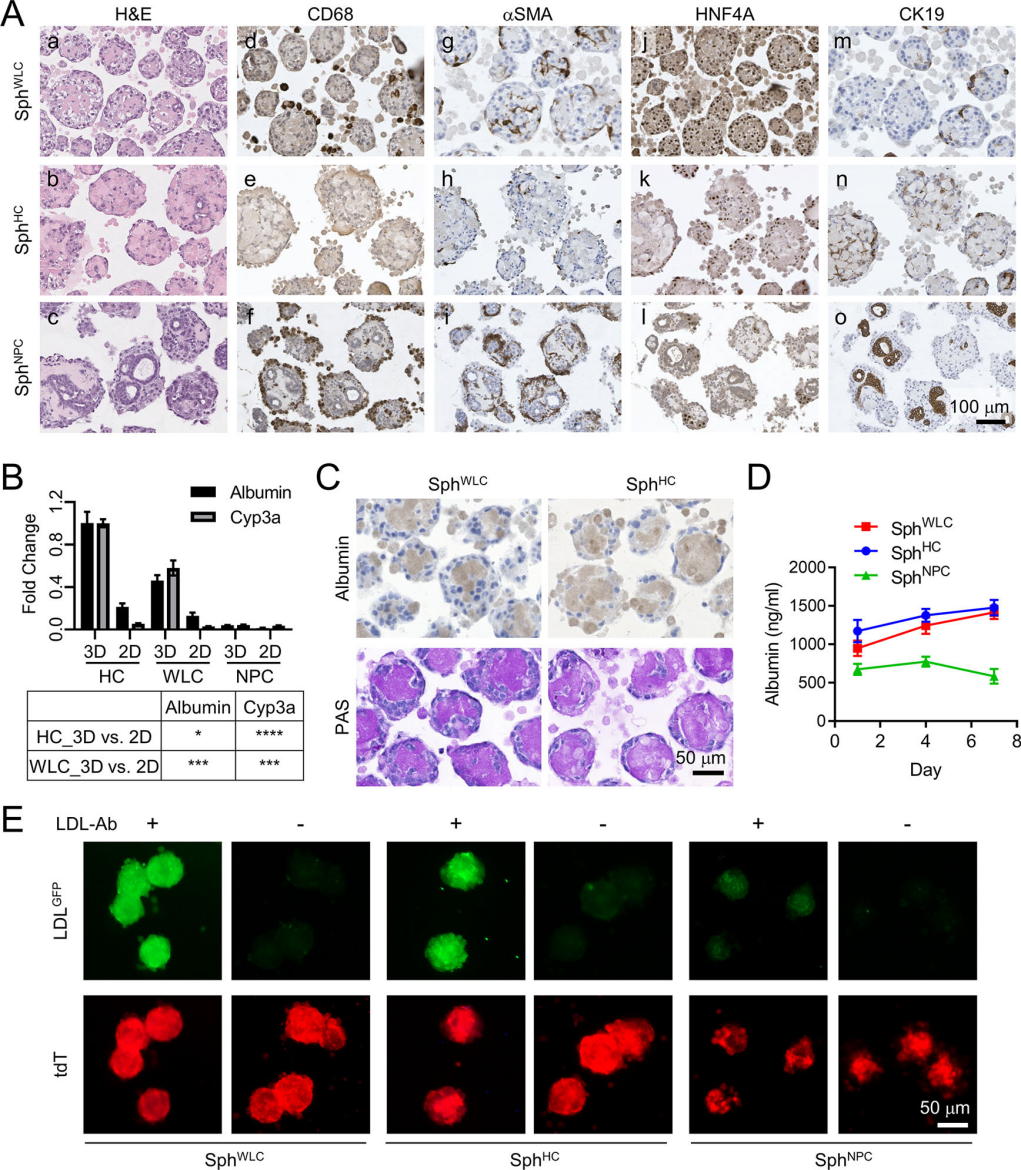
**Characterization of WLC, HC and NPC spheroids.** (A) Hematoxylin and Eosin (H&E) staining and CD68, αSMA, HNF4A and CK19 immunohistochemistry (IHC) of Day 7 WLC, HC and NPC spheroids for the indicated hepatic lineage markers. Scale bar: 100 µm. (B) Quantitative RT-PCR of Day 7 WLC, HC and NPC spheroids for the two indicated HC marker genes. Table below shows the unpaired two-tailed Student’s *t*-test comparison of the indicated culture conditions; *P<0.05, ***P<0.001, ****P<0.0001. (C) Albumin IHC and periodic acid–Schiff (PAS) staining of WLC spheroids (Sph^WLC^) and HC spheroids (Sph^HC^). Scale bar: 50 µm. (D) Albumin levels in the spheroid conditioned medium measured by enzyme-linked immunosorbent assay on Day 1, 4 and 7. A base level of albumin was detected in NPC spheroids (Sph^NPC^) because a 0.1% bovine serum albumin solution was used to dissolve the growth factors supplemented in the spheroid culture medium. (E) Low-density lipoprotein (LDL) uptake assay of the spheroids with (+) or without (−) anti-LDL antibody (LDL-Ab). Scale bar: 50 µm.

Sph^WLC^, Sph^HC^ and Sph^NPC^ were collected after 7 days in culture, paraffin processed, and underwent Hematoxylin and Eosin (H&E) and immunohistochemical characterization: alpha-smooth muscle actin (αSMA; also known as ACTA2) for activated HSCs, CD68 for activated Kupffer cells, HNF4A for HCs, and CK19 (also known as KRT19) for cholangiocytes and hepatic progenitor cells (HPCs) (Fig. 2A). Only rare endothelial cells were detected in Sph^WLC^ via CD31 (also known as PECAM1) and hepatic sinusoidal endothelial cell marker (HSEC) immunohistochemistry (IHC) (Fig. S2). Cholangiocytes are considered as NPCs in this study, although there are controversies regarding whether they are NPCs or liver parenchymal cells (Racanelli and Rehermann, 2006; Manco et al., 2018). As expected, NPCs with strong CD68 and αSMA positivity were found in Sph^WLC^ but not in Sph^HC^, both of which showed a higher content in Sph^NPC^ (Fig. 2A,d-i). Interestingly, the cellular organization of Sph^NPC^ was somewhat reminiscent of that of the liver sinusoids, with CD68^+^ Kupffer cells located in the outermost layer, αSMA^+^ HSCs in the middle layer and CK19^+^ cholangiocytes forming ducts in the inner layer (Fig. 2A,f).

Additional quantitative RT-PCR was performed for *Alb* (encoding albumin) and *Cyp3a* (encoding cytochrome P450, family 3, subfamily A), two HC marker genes, using spheroids collected after 7 days in culture. This confirmed the preservation of the differentiated features of HCs in the Sph^WLC^ comparable to Sph^HC^, whereas very low levels of both genes were detected in Sph^NPC^ (Fig. 2B). The presence of the NPC lineages in Sph^WLC^ may explain the lower levels of the two genes in these spheroids than in Sph^HC^. The same HCs and WLCs we put in the standard two-dimensional (2D) culture showed markedly lower levels of both genes than their spheroids, confirming 3D spheroids as a better *in vitro* model for preserving HC differentiation than 2D culture (Fig. 2B; Fig. S3). Further characterization of HCs with albumin IHC, periodic acid–Schiff (PAS) staining, albumin enzyme-linked immunosorbent assay (ELISA) and low-density lipoprotein (LDL) uptake assay confirmed the comparable, differentiated features of HCs in both Sph^WLC^ and Sph^HC^ (Fig. 2C-E). Interestingly, we noticed that, compared to Sph^WLC^, Sph^HC^ had a lower percentage of HNF4A^+^ HCs (Fig. 2A,j versus k; Fig. S4A). The majority of HCs in Sph^HC^ also showed a low level of CK19, which we did not observe in HCs in Sph^WLC^ (Fig. 2A,m versus n). In addition, we observed a small number of Sph^HC^ that were composed of predominantly CK19^+^ cells arranged into duct-like structures, which we did not notice in Sph^WLC^ (Fig. S4B,a-c). Compared to the CK19^+^ ducts in Sph^NPC^, these CK19^+^ ducts in Sph^HC^ were significantly more proliferative, as indicated by Ki67 (also known as MKI67) IHC (Fig. S4B,f,g versus h, Fig. S4C). These cells could be HCs dedifferentiating into regenerative HPC-like cells because HC dedifferentiation is commonly observed in their *in vitro* cultures (Guo et al., 2017; Schaub et al., 2018; Michalopoulos et al., 2002).

### NPCs, not HCs, suppress tumor cell proliferation in spheroid co-culture

**Fig. 3.**
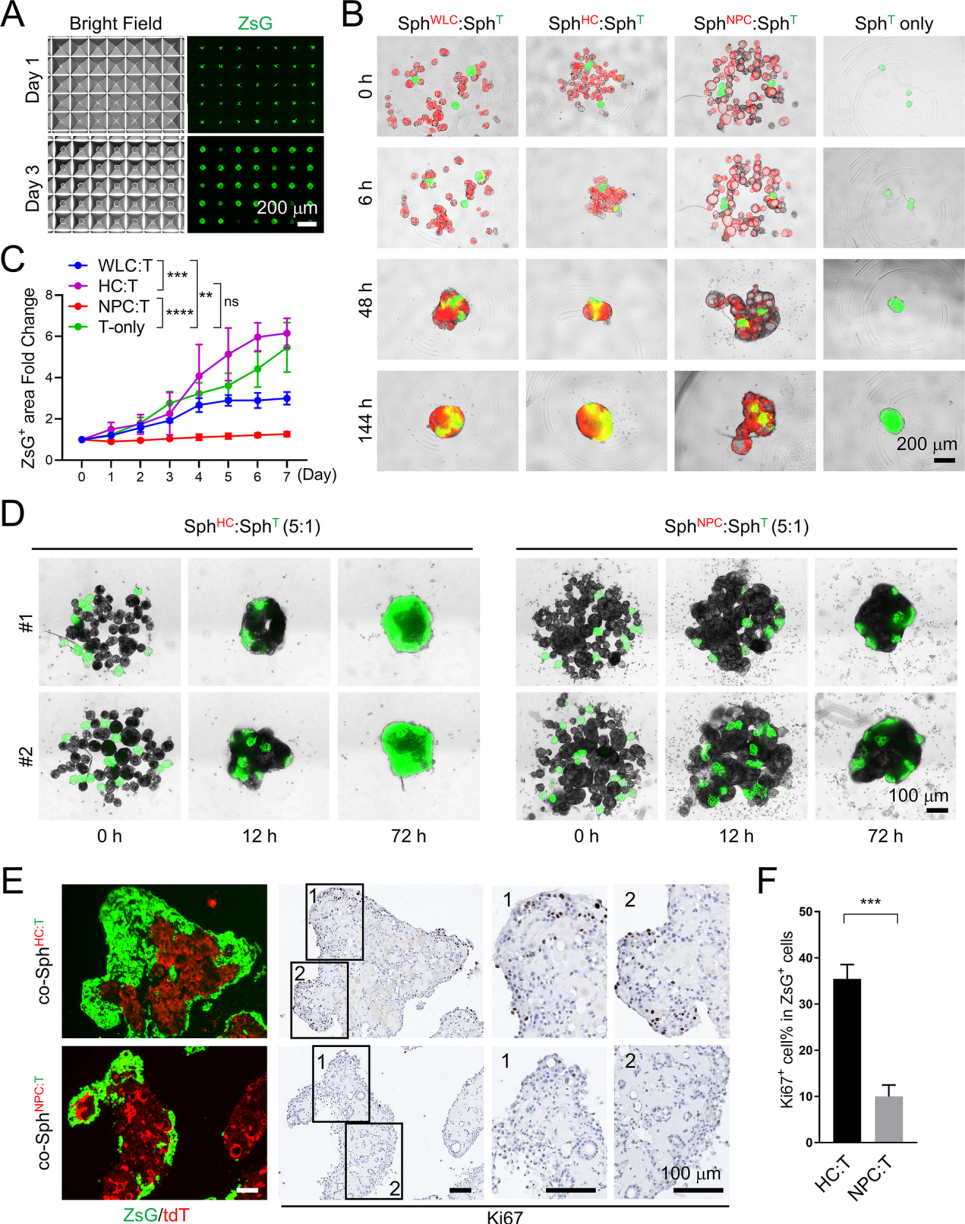
**NPC spheroids, not HC spheroids, suppress the growth of *Prom1^CreERT2^; Pten^flx/flx^; Tp53^flx/flx^; Rosa-ZsGreen* (PPTR) liver tumor cells in spheroid suspension co-cultures.** (A) Day 1 and Day 3 brightfield and ZsGreen (ZsG) fluorescence images of PPTR liver tumor spheroids in AW400 plates. Scale bar: 200 µm. (B) Merged ZsG/tdT images of the indicated spheroid co-cultures at the indicated times. Normal spheroids and tumor spheroids were mixed in a 15:1 ratio. Scale bar: 200 µm. (C) Fold change of the ZsG^+^ tumor area in the co-culture conditions from Day 0 to Day 7. Two-way ANOVA; **P<0.01, ***P<0.001, ****P<0.0001; ns, not significant. (D) ZsG images of Sph^HC^:Sph^T^ and Sph^NPC^:Sph^T^ co-cultures at the indicated times. Normal spheroids and tumor spheroids were mixed in a 5:1 ratio. Scale bar: 100 µm. (E) Merged ZsG/tdT images and Ki67 IHC of Sph^HC^:Sph^T^ co-spheroids (co-Sph^HC:T^) and Sph^NPC^:Sph^T^ co-spheroids (co-Sph^NPC:T^) collected after 7 days in co-culture. Scale bars: 100 µm. (F) Quantification of the Ki67^+^ cell content among the ZsG^+^ tumor cells in the co-Sph^HC:T^ and co-Sph^NPC:T^. Images are representative of three independent batches of spheroid co-culture. Unpaired two-tailed Student’s *t*-test; ***P<0.001.

As we were successful in generating large numbers of Sph^WLC^, Sph^HC^ and Sph^NPC^ using AggreWell microplates, we next tested their co-culture with mouse liver tumor spheroids (Sph^T^) as an attempt to model tumor–liver interaction *in vitro*. We have previously reported the establishment of multiple metastatic mouse liver cancer organoid lines from a PPTR mouse model of HCC (Zhu et al., 2016; Li et al., 2018). Upon serial orthotopic transplantation, tumors produced by PPTR organoids acquired increasing traits of iCCA (Li et al., 2018). Tumor organoids were similarly established from PPTR orthotopic iCCA tumors [PPTR intrahepatic cholangiocarcinoma organoids (iCCAOs)] (Marsee et al., 2021). However, PPTR iCCAOs were heterogeneous in size and shape (Fig. S5), which made it challenging to use them in quantitative assays. Therefore, we generated PPTR tumor spheroids using dissociated PPTR iCCAO single cells at 125 cells/microwell in AW400 microplates and the same organoid culture medium. We found that homogenous PPTR spheroids could be readily generated within 1-2 days and all PPTR Sph^T^ expressed strong ZsG fluorescence (Fig. 3A).

PPTR Sph^T^ were collected after 48 h and cultured alone or mixed with Sph^WLC^, Sph^HC^ or Sph^NPC^ at a ratio of 1:15, with approximately three Sph^T^ and 45 normal spheroids, in 96-well U-bottom microplates in suspension (Fig. 3B). We found that, in all four conditions, normal and tumor spheroids aggregated rapidly and formed one large co-spheroid by 48 h. After spheroid aggregation, the ZsG^+^ tumor area started to expand, indicating active tumor cell growth. We found that the ZsG^+^ tumor area expanded at a similar rate in Sph^T^-alone (co-Sph^T^) and Sph^HC^:Sph^T^ co-spheroids (co-Sph^HC:T^), slower in Sph^WLC^:Sph^T^ co-spheroids (co-Sph^WLC:T^) and the slowest in Sph^NPC^:Sph^T^ co-spheroids (co-Sph^NPC:T^) (Fig. 3B,C). To confirm the suppressive effect of NPCs on tumor cell growth, we repeated the Sph^HC^:Sph^T^ and Sph^NPC^:Sph^T^ co-cultures with an increased tumor:normal spheroid ratio of 1:5, mixing approximately ten Sph^T^ with 50 Sph^HC^ or Sph^NPC^ per well. Again, we observed a significantly slower expansion of the ZsG^+^ tumor area in co-Sph^NPC:T^ than in co-Sph^HC:T^ over the 7 days we maintained the co-cultures (Fig. 3D). Ki67 IHC confirmed a much lower proliferation rate of the ZsG^+^ tumor cell population in co-Sph^NPC:T^ than in co-Sph^HC:T^ (Fig. 3E,F). These results indicate that peritumoral NPCs are able to suppress tumor growth, an ability we did not find with HCs we similarly placed peritumorally.

### Tumor–liver interaction induces activation and accumulation of HSCs, which are tumor suppressive and hepatoprotective, in spheroid co-culture

**Fig. 4.**
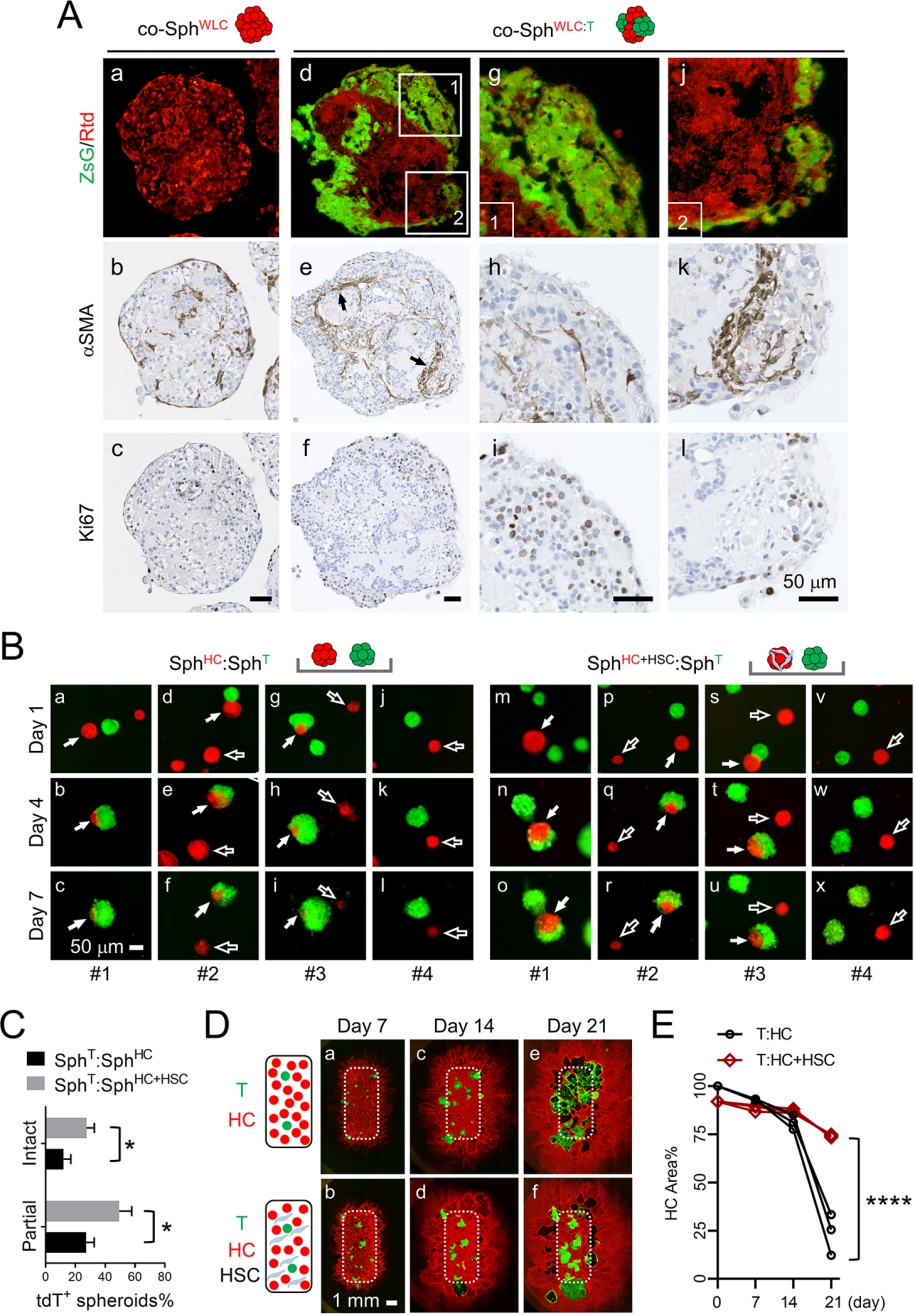
**Activation and a hepatoprotective role of hepatic stellate cells (HSCs) in tumor–liver interaction.** (A) ZsG/tdT images and IHC of αSMA and Ki67 in co-Sph^WLC^ and co-Sph^WLC:T^ sections. (g-i) Enlarged images of the boxed Area 1 in d; (j-l) enlarged images of the boxed Area 2 in d. Scale bars: 50 µm. (B) ZsG/tdT images of the indicated two spheroid co-cultures on Day 1, 4 and 7. Four examples are shown in each condition. Images in the same column are taken from the same areas of the co-cultures. Filled arrows indicate tdT^+^ Sph^HC^ and Sph^HC+HSC^ in direct contact with ZsG^+^ Sph^T^; open arrows indicate tdT^+^ Sph^HC^ and Sph^HC+HSC^ that are not in contact with ZsG^+^ Sph^T^. Scale bar: 50 µm. (C) Quantification of the percentage of partially ‘eaten’ and intact tdT^+^ spheroids on Day 7 compared to Day 1 in each co-culture condition. Counting was performed from three independent batches. Unpaired two-tailed Student’s *t*-test; *P<0.05. (D) Schematic representation and ZsG/tdT images of T:HC and T:HC+HSC 2D co-cultures on Day 7, 14 and 21. Dotted line boxes indicate the original seeding area within the chamber insert. Scale bar: 1 mm. (E) Percentage of the area occupied by tdT^+^ HCs within the boxed area in D on Day 7, 14 and 21 in comparison to Day 1. Images are representative of three independent batches of spheroid and 2D co-culture. Two-way ANOVA; ****P<0.0001.

To characterize potential responses from NPCs upon their interaction with tumor cells, we compared the αSMA, CD68 and CK19 IHC of 7-day co-Sph^WLC:T^ with that of 7-day co-Sph^WLC^ aggregated in suspension without Sph^T^. Among the three NPC markers, αSMA showed the most obvious tumor-associated changes (Fig. 4A; Fig. S6). Compared to the random distribution of a small number of αSMA^+^ HSCs in the co-Sph^WLC^, there was a marked accumulation of αSMA^+^ HSCs at the Sph^WLC^:Sph^T^ interface in co-Sph^WLC:T^ (Fig. 4A,b versus e). This tumor–WLC interaction-induced HSC accumulation on their interface was confirmed using a second PPTR tumor cell line (PPTR-2) derived from a different iCCA orthotopic tumor (Fig. S7). Tumor cells next to the areas with more HSC accumulation tended to be less proliferative, indicated by Ki67 IHC (Fig. 4A,h,i versus k,l; Fig. S8), suggesting a growth-suppressive role of peritumoral HSCs.

To directly assess the potential tumor-suppressive role of HSCs, we generated spheroids with 1:1 mixed HCs and HSCs (Sph^HC+HSC^). Mouse primary HSCs were purchased due to their low number in freshly isolated liver cells and were activated via standard 2D culture in plastic culture plates for one to two passages (Mederacke et al., 2015). The presence of activated HSCs in Sph^HC+HSC^, but not in Sph^HC^, was confirmed by αSMA IHC (Fig. S9). We then seeded Sph^T^ with Sph^HC^ or Sph^HC+HSC^, at a 4:1 ratio, onto a flat-bottom 24-well microplate precoated with 100% Matrigel and monitored their interaction over 7 days via fluorescence microscopy. We noticed a similar aggregation of the spheroids within 2 days, although mostly limited between two to three spheroids within a close range (Fig. 4B). We found that the tdT fluorescence of Sph^HC^ was quickly diminished when they were in physical contact with Sph^T^ (Fig. 4B, filled arrows in a-i), indicating a tumor-induced HC death. This elimination of HCs by tumor cells was significantly reduced in the Sph^T^:Sph^HC+HSC^ co-culture (Fig. 4B, filled arrows in m-u). Even for the normal spheroids that were not in direct contact of Sph^T^, we noticed that Sph^HC^ were dying faster than Sph^HC+HSC^ (Fig. 4B, open arrows in d-l versus p-x). At the end of the Day 7, there was a significantly higher percentage of partially ‘eaten’ as well as intact Sph^HC+HSC^ remaining than Sph^HC^ (Fig. 4C). Partially ‘eaten’ and intact Sph^HC+HSC^ were defined by a reduction in their tdT^+^ area from Day 1 to Day 7 of more than 50% and less than 10%, respectively. To further validate this hepatoprotective role of HSCs, we employed a more quantitative co-culture method using primary tdT^+^ HCs that had been adopted into 2D culture via continuous passaging up to five passages. HCs were then seeded into a chambered culture insert alone or with 20% HSC added. The total number of cells seeded remained the same. Twenty PPTR tumor cells were mixed into each condition. We monitored tumor cell growth and HC death via fluorescence microscopy over 21 days (Fig. 4D,E) and, once again, found a significantly slower reduction in the tdT^+^ HC area in T:HC+HSC co-culture than in T:HC co-culture. These results together revealed a hepatoprotective role of peritumoral HSCs to HCs.

### Tumor development induces HSC activation at the tumor border in mouse and patient liver

**Fig. 5.**
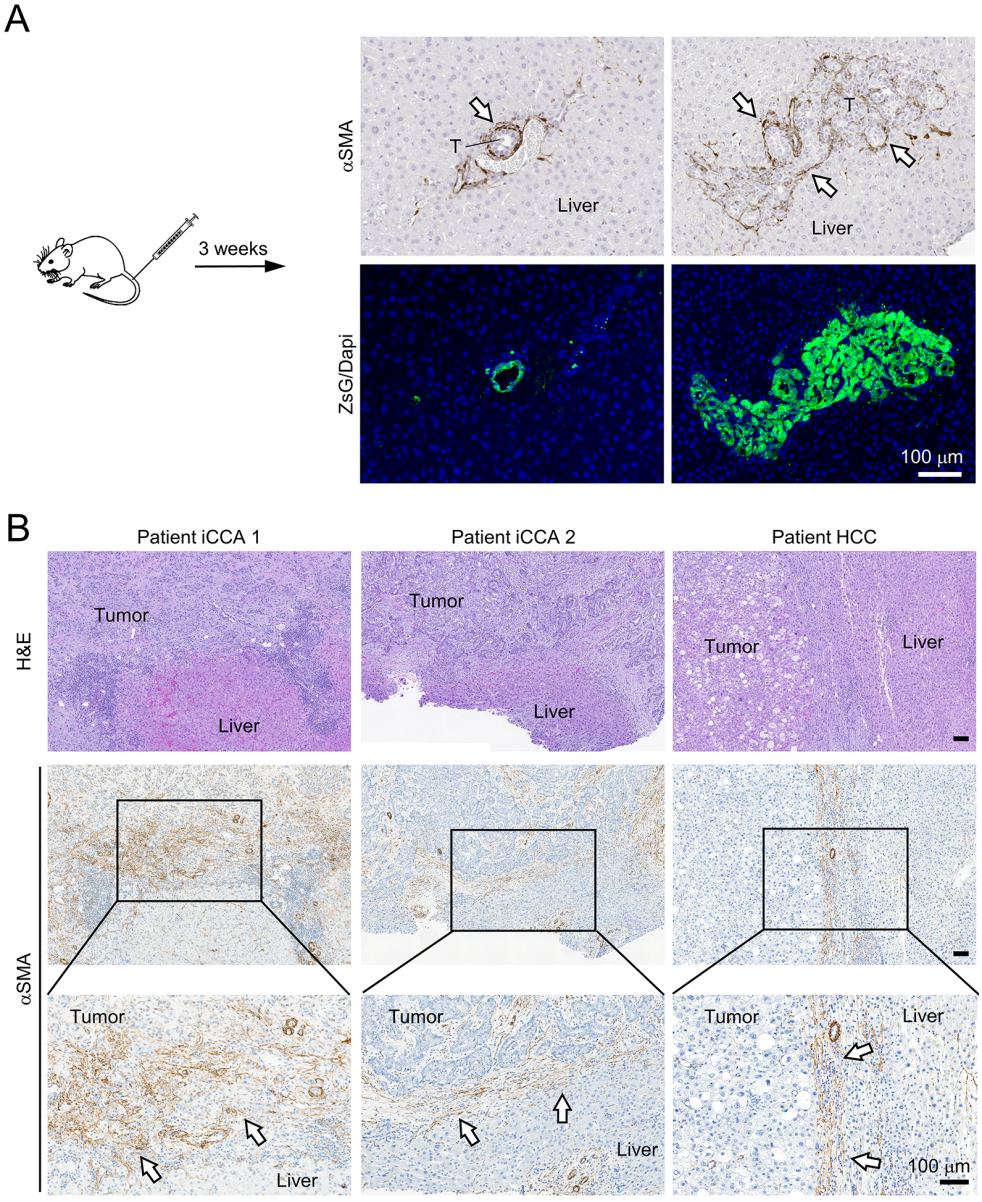
**Peritumoral HSC activation in early-stage mouse and patient liver tumors.** (A) Images of αSMA IHC and matching ZsG/DAPI fluorescence of 3-week PPTR liver tumors generated via tail vein injection. Arrows indicate αSMA^+^ cells at the tumor border. T, tumor. Scale bar: 100 µm. Images are representative of tumors from five mice. (B) H&E staining and αSMA IHC of two intrahepatic cholangiocarcinoma (iCCA) and one hepatocellular carcinoma (HCC) early-stage tumors from patients. Arrows indicate αSMA^+^ cells at the tumor border. Scale bars: 100 µm.

To validate whether the HSC activation we observed in the Sph^WLC^ co-cultured with Sph^T^ recapitulated the liver's response to tumorigenesis at the early stage, we transplanted PPTR tumor cells via tail vein injection into CD-1 Nu/Nu mice and examined the early tumor development in the liver after 3 weeks (Fig. 5A). We found that this was an efficient way to generate and capture early-stage liver tumors. IHC analysis revealed evident accumulation of αSMA^+^ HSCs at the tumor border (Fig. 5A). No αSMA^+^ HSCs were found in the liver areas bearing no tumors, indicating that the αSMA^+^ HSCs were tumor activated. We also acquired three early-stage patient tumors, including two iCCA and one HCC that were resected without neoadjuvant treatment. We found similar accumulation of αSMA^+^ HSCs at the tumor border (Fig. 5B). HSCs in the two iCCA cases had signs of tumor infiltration in certain areas, which was expected considering the aggressive nature of this type of primary liver cancer. Overall, HSC activation and accumulation at the tumor border in both early-stage mouse and patient liver tumors similar to that in the co-Sph^WLC:T^ indicated that this spheroid co-culture was capable of modeling *in vivo* response of the liver to tumorigenesis.

## DISCUSSION

In this study, we reported the generation of hepatic spheroids from freshly isolated mouse WLCs, HCs and NPCs at high efficiencies using AggreWell microplates. AggreWell microplates have previously been used to establish spheroids from rat HCs but not yet been tested in the mouse system (Ijima et al., 2007; Ha et al., 2019). Compared to the conventional 96-well microplate-based liver spheroid culture that requires 1000-1500 HCs per well (Bell et al., 2016; Nautiyal et al., 2021; Kyffin et al., 2019), this method requires much fewer HCs to generate a large number of homogeneous spheroids for downstream investigations. Interestingly, although WLCs and HCs formed spheroids similarly under our experimental conditions, we found that HCs in the Sph^WLC^ maintained their differentiation features better than HCs in Sph^HC^. Compared to Sph^WLC^, Sph^HC^ exhibited signs of HC dedifferentiation, with increased *CK19* expression and proliferation during the 7 days of culture, which model the original tumor less accurately. HC dedifferentiation is common in HC 2D and 3D *in vitro* models, which has posed challenges to using these models to understand valid HC biology and pathology (Guo et al., 2017; Michalopoulos et al., 2002). Our observations of HCs being more prone to dedifferentiation in the absence of NPCs suggests a potential stabilization of HC differentiation by NPCs in liver homeostasis, yielding a more accurate model system.

NPC spheroids have not yet been reported. We found that 2000 cells/microwell in AW800 plates is an optimal condition to form Sph^NPC^. Cholangiocytes, HSCs and Kupffer cells were preserved in Sph^NPC^ and showed an organization somewhat similar to that of the liver sinusoids *in vivo*. We did not detect endothelial cells in Sph^NPC^. Hepatic endothelial cells are known to be challenging to isolate and culture (Poisson et al., 2017). Further optimization will be required to allow the integration of this important NPC lineage into Sph^WLC^.

Our co-culture of normal liver spheroids and PPTR liver tumor spheroids, to our knowledge, is one of the first attempts to model tumor–liver interaction *in vitro*. Tumor–liver interaction can be difficult to dissect in a complex *in vivo* setting when different hepatic and non-hepatic cellular players are constantly communicating and coordinating. *In vitro* models, on the other hand, can be complementary tools to dissect tumorigenesis and the host response under different conditions. We found that Sph^HC^ had little effect on Sph^T^ growth upon their aggregation and, in fact, can be quickly eliminated by Sph^T^. In contrast, Sph^WLC^ and Sph^NPC^ exhibited the ability to effectively suppress tumor cell proliferation. We then showed that, among the NPC lineages that were preserved in Sph^WLC^, HSCs had a particularly strong response to tumor interaction by rapid activation and accumulation at the tumor–WLC spheroid interface, a phenomenon we confirmed in early-stage mouse and patient liver tumors. Mixing HSCs into HCs provided strong protection over tumor-induced HC death, supporting a hepatoprotective role of HSCs when activated in the peritumoral TME.

Changes in the NPC populations within the tumor mass have been reported in liver cancer patients, and studies have predominantly shown that these changes are protumorigenic and prometastatic (Ji et al., 2015; Budhu et al., 2006; Zhu et al., 2008; Yang et al., 2011; Yin et al., 2013; Song et al., 2016). However, NPC activation in the tumor-adjacent liver is less studied. Based on our study, we propose that NPC-associated changes in the peritumoral liver, especially the rapid activation of HSCs at the tumor border, are a defensive mechanism of the non-malignant liver to retard tumor growth and protect HCs. Our study shows that HCs are a vulnerable cell type that can be quickly eliminated when in direct contact with tumor cells. The barrier established by activated NPCs between tumor and the surrounding liver can be a critical protection over tumor-induced HC death. Indeed, recent studies have started to argue that cirrhosis, a late-stage liver fibrosis condition that heavily involves NPC activation, may be a liver-protective response rather than a risk factor for liver cancer (Garrido and Djouder, 2021). Studies in other solid tumors, particularly in pancreatic cancer, have also shown that deleting myofibroblasts can accelerate tumor progression (Rhim et al., 2014; Özdemir et al., 2014; Chen et al., 2021), further supporting a tumor-restraining role of myofibroblasts under certain conditions. Besides HSCs, other NPC lineages, especially immune cells such as Kupffer cells, have been found to be enriched at the tumor border in the adjacent liver tissue (Carapeto et al., 2022) and possibly contribute to a similar hepatoprotective response of the liver to tumorigenesis to a certain extent. We did observe a better preservation of CD68^+^ Kupffer cells in co-Sph^WLC:T^ than in co-Sph^WLC^, although these cells were not particularly enriched at the WLC:T spheroid interface. The known involvement of HSCs in liver immune microenvironment further complicates their crosstalk with hepatocytes and other immune populations in liver cancer (Wang et al., 2022; Coulouarn et al., 2012). Future studies are in line to investigate the impact of the individual NPC lineages on tumor–liver interaction using the same *in vitro* and *in vivo* models in this study. Further investigations are also needed to identify specific molecular mechanisms accounting for the phenotypic changes we observed in this study. Overall, our study suggests an interesting hepatoprotective role of peritumoral NPCs in liver tumorigenesis, and shows that the *in vitro* spheroid co-culture can be a useful model system to track and dissect tumor–liver interaction.

## MATERIALS AND METHODS

### Mice and tail vein injection

*B6.129(Cg)-Gt(ROSA)26Sor^tm4(ACTB-tdTomato,-EGFP)Luo^/J* (mTmG) mice were purchased from The Jackson Laboratory (Strain #007676; Bar Harbor, ME, USA). Two-month-old mTmG male and female mice were used for liver cell isolation and spheroid generation. No sex-specific differences were noted. PPTR tumor cells were cultured as previously described (Li et al., 2018), and injected into the male and female *Crl:CD1-Foxn1^nu/nu^* (CD-1 nude) mice (Strain Code 086, Charles River Laboratories, Wilmington, MA, USA) via the tail vein at 1×10^6^ cells/mouse in 100 µl PBS. All mice were maintained in the Animal Resource Center at St. Jude Children's Research Hospital. Mice were housed in ventilated, temperature- and humidity-controlled cages under a 12-h light/12-h dark cycle and given a standard diet and water *ad libitum*. Animal protocols were approved by the St. Jude Animal Care and Use Committee, and the care and use of experimental animals complied with relevant local animal welfare laws, guidelines and policies.

### Primary mouse liver cell isolation

Liver cells were isolated from 2-month-old mTmG mouse liver using the standard two-step collagenase perfusion method (Berry and Friend, 1969; Li et al., 2010). Briefly, mice were anesthetized with isoflurane, and the liver was perfused with 30 ml prewarmed EGTA solution (0.5 mM) through the inferior vena cava for 10 min, and perfused with 30 ml prewarmed pronase solution (14 mg/mouse; Sigma-Aldrich, St Louis, MO, USA), followed by 30 ml collagenase solution (3.7 U/mouse; Roche, Basel, Switzerland) for 10 min each. The liver was then removed and transferred to a sterile Petri dish and gently minced with forceps. The liver was further digested with the pronase/collagenase solution with 1% DNase I (Sigma-Aldrich) for 25 min at 40°C. Cells were filtered by a 70-μm filter and used directly as unfractionated WLCs, or centrifugated at 50 ***g*** for 5 min to pellet HCs. The supernatant was then centrifuged at 580 ***g*** for 5 min to pellet NPCs. WLCs, HCs and NPCs were washed twice in cold Advanced DMEM/F12 (Thermo Fisher Scientific, Waltham, MA, USA) and subjected to spheroid and 2D culture.

### Mouse hepatic spheroid and organoid culture

WLCs, HCs and NPCs were seeded in 24-well AW400 culture plates (STEMCELL Technologies, Vancouver, Canada) at 125, 250, 500 and 1000 cells/microwell, and 24-well AW800 plates (STEMCELL Technologies) at 500, 1000 and 2000 cells/microwell, for 7 days according to the manufacturer's instructions. PPTR tumor cells were seeded at 125 cells/microwell in AW400 plates and cultured for 2 days. The same culture medium was used for all spheroid cultures: 50% mouse HepatiCult™ Organoid Growth Medium (STEMCELL Technologies), 50% cholangiocyte organoid culture medium reported previously (Hu et al., 2018) and 1% Matrigel. The cholangiocyte organoid culture medium was prepared by supplementing advanced DMEM/F12 (Thermo Fisher Scientific) with 50% conditioned medium from L-WRN cells (CRL-3276™, American Type Culture Collection, Manassas, VA, USA), 10 mM HEPES, 1% GlutaMax (Thermo Fisher Scientific), 1% penicillin–streptomycin (Thermo Fisher Scientific), 2% B27 (Thermo Fisher Scientific), 1% N2 (Thermo Fisher Scientific), 3 μm CHIR99021 (Sigma-Aldrich), 1.25 mM N-acetylcysteine (Sigma-Aldrich), 10 mM nicotinamide (Sigma-Aldrich), 10 nM recombinant gastrin (Sigma-Aldrich), 50 ng/ml EGF (Peprotech), 50 ng/ml FGF7 (Peprotech), 50 ng/ml FGF10 (Peprotech), 25 ng/ml HGF (Peprotech), 1 μM A83-01 (Tocris, Bristol, UK), 10 μM Rho Inhibitor γ-27632 (STEMCELL Technologies) and 1% growth factor-reduced Matrigel. The same cholangiocyte organoid culture medium was used to culture PPTR tumor organoids.

### Mouse spheroid co-culture

Sph^WLC^, Sph^HC^ and Sph^NPC^ spheroids were collected by low-speed centrifugation on Day 7, mixed with Day 3 tumor spheroids at a ratio of 15:1 or 5:1 in 96-well U-bottom microplates, and cultured for 7 days with the same culture medium as above. All cultures were kept under continuous shaking at 80 rpm on a CO_2_-resistant orbital shaker (Thermo Fisher Scientific). To generate Sph^HC+HSC^, freshly isolated HCs from mTmG mice were mixed with primary mouse HSCs (ScienCell Research Laboratories, Carlsbad, CA, USA) at a ratio of 4:1 (HC:HSC), and the mixture was then seeded into AW400 plates at 250 cells/microwell to generate spheroids.

### LDL uptake assay

An LDL uptake assay (ab133127, Abcam, Cambridge, UK) was performed according to the manufacturer's instructions. Approximately 20 Sph^WLC^, Sph^HC^ and Sph^NPC^ were transferred from the AggreWell plate to a 96-well flat-bottom microplate and allowed to attach for 1 h before proceeding with the experiment.

### Mouse albumin ELISA

Conditioned medium from the Sph^WLC^, Sph^HC^ and Sph^NPC^ cultures was collected at Day 1, 4 and 7. The conditioned medium was centrifuged at 3000 ***g*** for 10 min, and the supernatant was subjected to albumin detection with the Albumin mouse ELISA kit (ab108792, Abcam) according to the manufacturer's instructions.

### PAS staining

Four-micrometer paraffin sections of Sph^WLC^ and Sph^HC^ were prepared and subjected to standard PAS staining. Briefly, the sections were deparaffinized and oxidized in 0.5% periodic acid solution for 5 min, washed three times in distilled water and incubated with Schiff's reagent for 15 min. Sections were then washed three times in distilled water and counterstained using Mayer's Hematoxylin.

### Mouse liver cell 2D culture and co-culture

For the 2D culture of freshly isolated WLCs, HCs and NPCs, WLCs, HCs and NPCs were seeded at 1.5×10^5^/well, 1.5×10^5^/well and 1.2×10^6^/well, respectively, in six-well multiwell plates and cultured using the same medium used in spheroid culture. For HC+HSC:tumor 2D co-culture, a 24-well microplate was precoated with 1% Matrigel, and a 5×8.4 mm chamber insert (ibidi, Gräfelfing, Germany) was placed in the well. Twenty PPTR tumor cells were mixed with 1×10^4^ cells of HCs only or a 4:1 mixture of HCs and HSCs and seeded into the chamber insert. Cells were washed, and the insert was removed after 24 h.

### Quantitative RT-PCR

The total RNA in cells and spheroids was extracted using an RNeasy^®^ Mini Kit (Qiagen, Hilden, Germany). SuperScript^®^ III First-Strand Synthesis SuperMix for qRT-PCR (Invitrogen) was used for cDNA synthesis from 500 ng total RNA. FastStart Universal SYBR^®^ Green Master (ROX) (Roche) was used to perform the quantitative PCR assay. The results were analyzed using the 2^−ΔΔCt^ method, with *Actb* (β-actin) as the internal reference gene. Quantitative RT-PCR was performed using three batches of independent 3D and 2D cultures, and the results were plotted using GraphPad Prism 9.4. The PCR primers were as follows: *Alb*-F, 5′-GCGACTATCTCCAGCAAACT-3′; *Alb*-R, 5′-CACTTCCTGGTCCTCAACAA-3′; *Cyp3a*-F, 5′-ACCACCAGTAGCACACTTTC-3′; *Cyp3a*-R, 5′-CCAGGTATTCCATCTCCATCAC-3′; *Actb*-F, 5′-GTTGTCGACGACCAGCG-3′; *Actb*-R, 5′-GCACAGAGCCTCGCCTT-3′.

### Microscopy and GFP quantification

All co-cultures were monitored daily using an ECLIPSE Ts2R fluorescence microscope (Nikon, Tokyo, Japan). Daily ZsG/tdT fluorescence images of the spheroid suspension co-cultures were captured by NIS-Elements (Nikon). The ZsG^+^ tumor cell area in their co-cultures was measured by ImageJ, normalized to Day 0 and plotted using GraphPad Prism 9.4. ZsG/tdT fluorescence images of the 2D co-culture of HC:tumor and HC+HSC:tumor were captured by an ECLIPSE Ts2R fluorescence microscope on Day 1, 4 and 7. Day 1 and Day 7 images were used to count and determine the percentage of partially ‘eaten’ and intact tdT^+^ spheroids. Specifically, the tdT fluorescence area of individual normal spheroids on Day 1 to Day 7 was measured using ImageJ, and partially ‘eaten’ and intact spheroids were defined as those with more than 50% and less than 10% reduction in their tdT^+^ area, respectively. ZsG/tdT fluorescence images of the 2D co-culture of HC:tumor and HC+HSC:tumor were captured every 7 days by a FX Automated Microscope (Lionheart, Winooski, VT, USA) until Day 21. tdT^+^ HC area within the original seeding region was measured in ImageJ, normalized to Day 1 and plotted using GraphPad Prism 9.4. All measurements were taken from three independent co-cultures.

### IHC and quantification

Liver spheroids and tissues were fixed in neutral buffered formalin for 1 day at room temperature and submitted to HistoWiz Inc. (Brooklyn, NY, USA) for paraffin processing and sectioning. Paraffin sections (4 µm) were analyzed by direct fluorescence microscopy, H&E staining and IHC. IHC antibodies included anti-HNF4α (PP-K9218-00, R&D Systems, Minneapolis, MN, USA; 1:125); anti-αSMA (ab124964, Abcam; 1:1000); anti-Ki67 (ab16667, Abcam, Cambridge, MA, USA; 1:200); anti-CK19 (ab133496, Abcam; 1:250); anti-CD68 (ab125212, Abcam; 1:1000); anti-CD31 (ab12443, Abcam; 1:1000), anti-albumin (ab192603, Abcam; 1:50) and anti-HSEC (NB110-68095, Novus Biologicals, Centennial, CO, USA; 1:250). For the co-spheroids, direct ZsG and tdT fluorescence images were taken from sections prior to IHC. HNF4A^+^ and Ki67^+^ counting was performed on three spheroids each from three independent batches of spheroids and plotted using GraphPad Prism 9.4.

### iCCA patient samples

The de-identified iCCA patient tumor samples were obtained under a protocol approved by the Institutional Review Boards at St. Jude Children's Research Hospital and The University of Tennessee Health Science Center. Informed consent was obtained from sample donors. All clinical investigation related to sample collection was conducted according to the principles expressed in the Declaration of Helsinki.

### Statistics

Unpaired two-tailed Student's *t*-test was performed in GraphPad Prism 9.4 to compare two independent pairs of groups. Two-way ANOVA was performed when two or more groups were compared. *P*≤0.05 was considered statistically significant.

## Supplementary Material

10.1242/dmm.049750_sup1 Supplementary information

## Data Availability

All relevant data can be found within the article and its supplementary information.
