# Supplementary material for: A hepatoprotective role of peritumoral non-parenchymal cells in early liver tumorigenesis
Source: Dis Model Mech. 2023 Mar 7;16(3):dmm049750. doi: 10.1242/dmm.049750 (PMC10040241; doi:10.1242/dmm.049750)
Supplement: Supplementary information [file dmm-16-049750-s1.pdf]

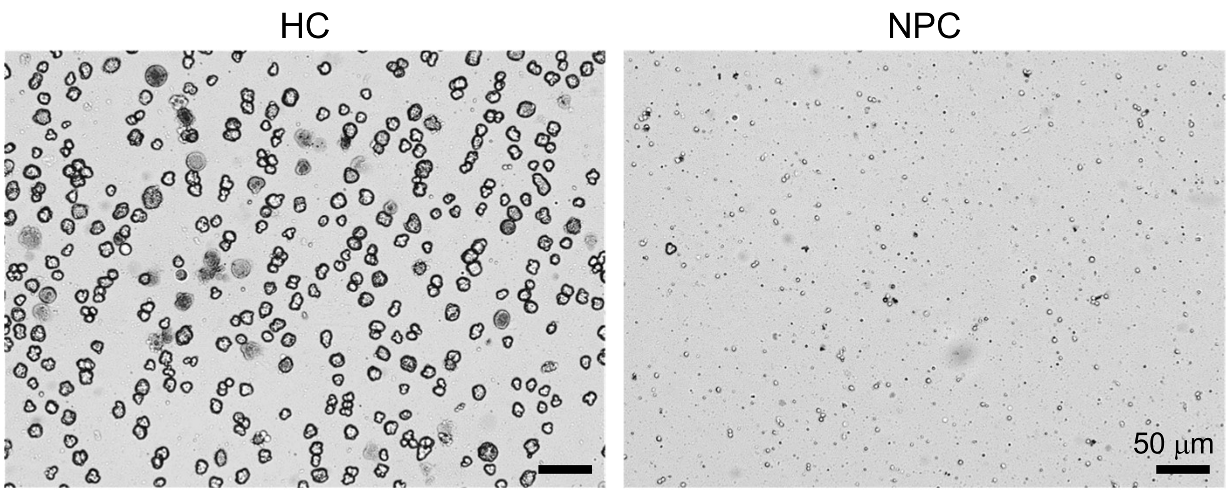

Fig. S1. The bright-field images of the HCs and NPCs freshly isolated from a mTmG mouse liver after gravity segregation.

Disease Models & Mechanisms • Supplementary information

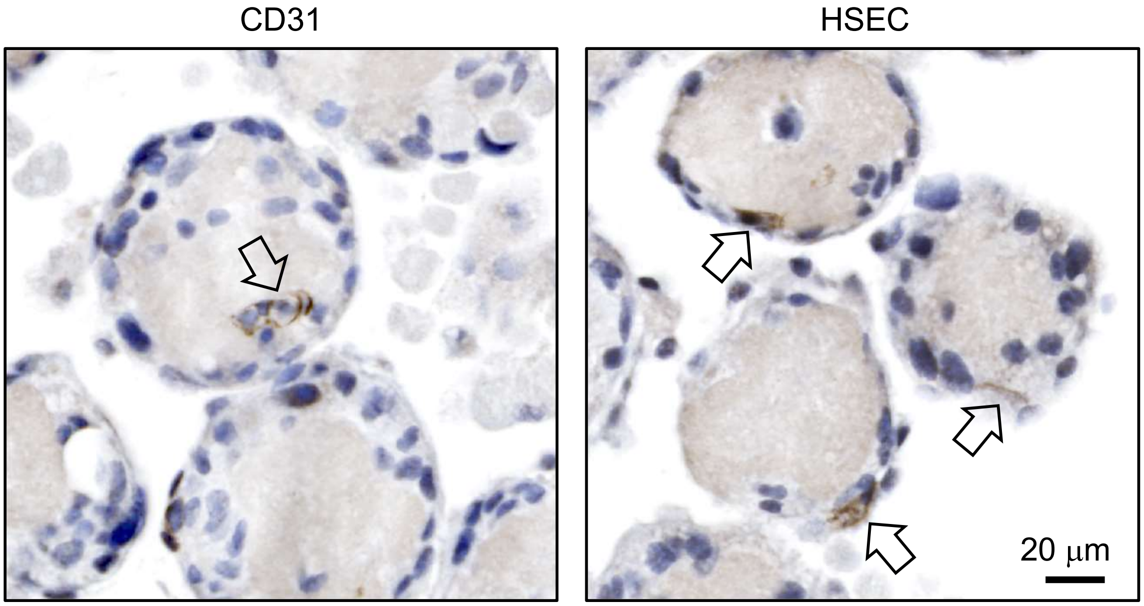

Fig. S2. Limited endothelial cells are present in Sph^{WLC}. CD31 and HSEC (hepatic sinusoidal endothelial cell marker) IHC of Sph^{WLC}. Arrows: Cells stained positive for the indicated marker.

Disease Models & Mechanisms • Supplementary information

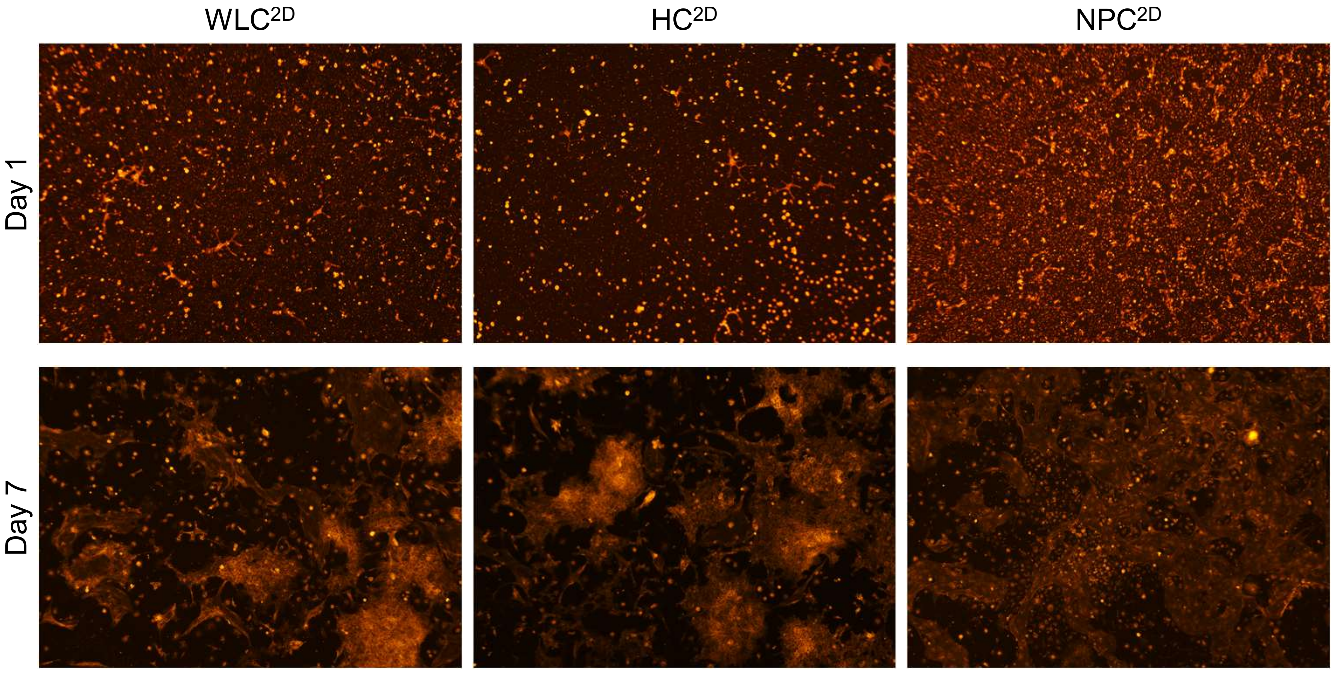

Fig. S3. Standard 2D culture of freshly isolated mTmG WLCs, HCs and NPCs. The tdT fluorescence images of the 2D cultured WLCs, HCs and NPCs on Day 1 and Day 7.

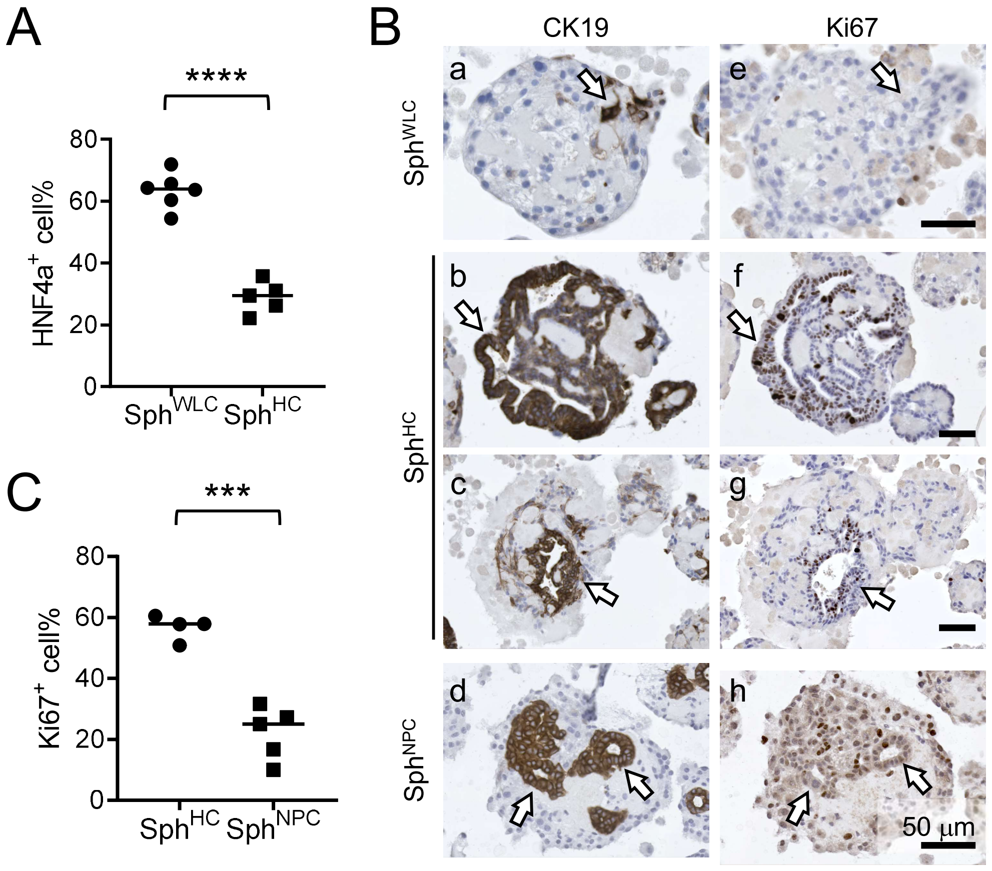

Fig. S4. Potential HC dedifferentiation in Sph^{HC}.

(A) Quantitative comparison of the HNF4a^{+} cell content of the WLC and HC spheroids. Student *t* test.

(B) IHC images of the indicated markers on the serial sections of Day 7 WLC, HC, and NPC spheroids. Open arrows: CK19^{+} structures in each spheroid type. Images on the same row share the same 50 mm scale bar.

(C) Quantitative comparison of the Ki67^{+} cell content of the CK19^{+} ducts in the HC and NPC spheroids.

Images are representative of three independent batches of spheroid culture. Student *t* test; *P* value: * <0.05, *** <0.001, **** <0.0001.

Disease Models & Mechanisms • Supplementary information

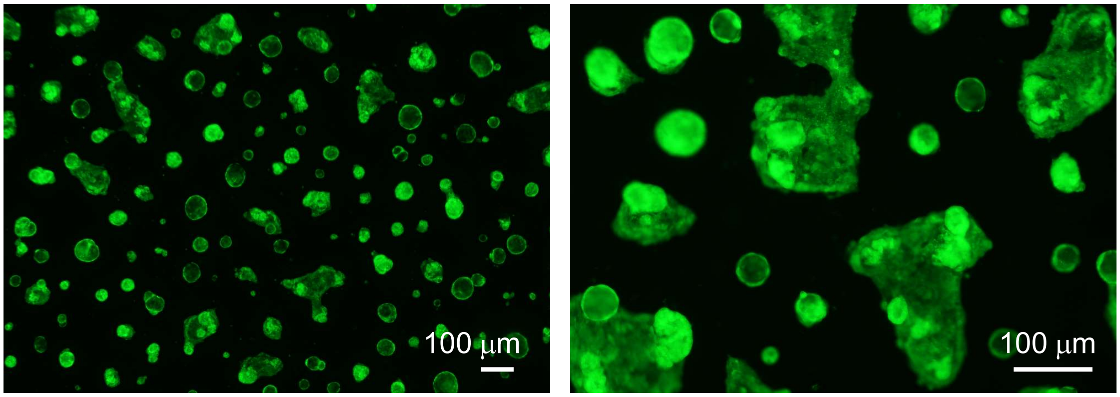

Fig. S5. PPTR tumor organoids are heterogeneous in size and shape.
The ZsG fluorescence images of PPTR tumor organoids on Day 3.

Disease Models & Mechanisms • Supplementary information

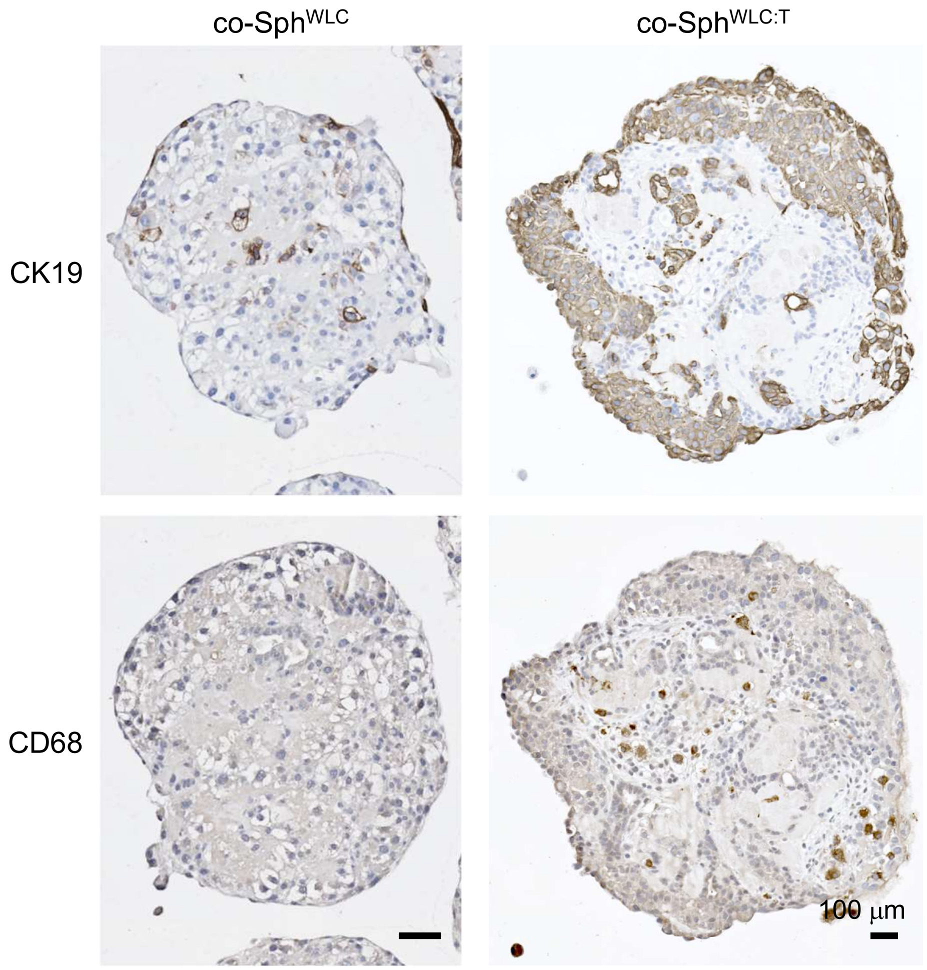

Fig. S6. Cholangiocytes and Kupffer cells showed limited response to tumor cell attachment in spheroid coculture.

CK19 and CD68 IHC on serial sections of co-Sph^{WLC} and co-Sph$^{WLC:T}$. CK19 positively was mainly found in the tumor cells in co-Sph$^{WLC:T}$. limited CD68^{+} Kupffer cells were present in co-Sph$^{WLC:T}$ but not in co-Sph^{WLC}.

Disease Models & Mechanisms • Supplementary information

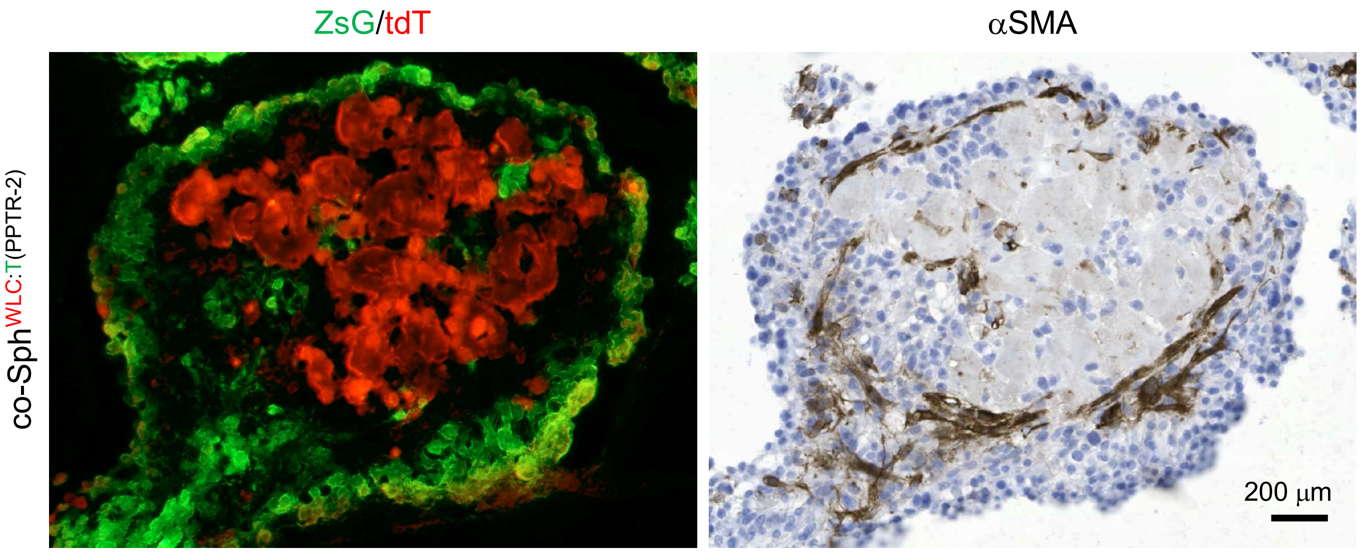

Fig. S7. PPTR-2 spheroids cocultured with Sph^{WLC} induce similar HSC accumulation at the spheroid interface.

ZsG/tdT and αSMA IHC images of Co-Sph$^{WLC:T}$ using PPTR-2 tumor cell line.

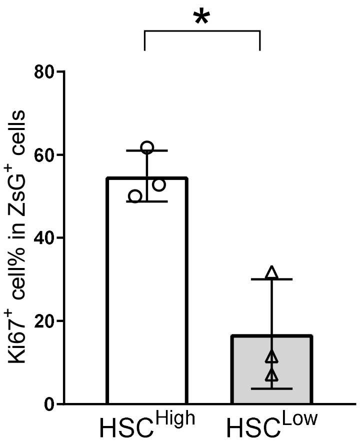

Fig. S8. Quantification of Ki67$^+$ cells% among the ZsG$^+$ tumor cells from HSC^{High} and HSC^{Low} areas in co-Sph$^{WLC:T}$. Statistics: Student t test, *P* value, * < 0.05.

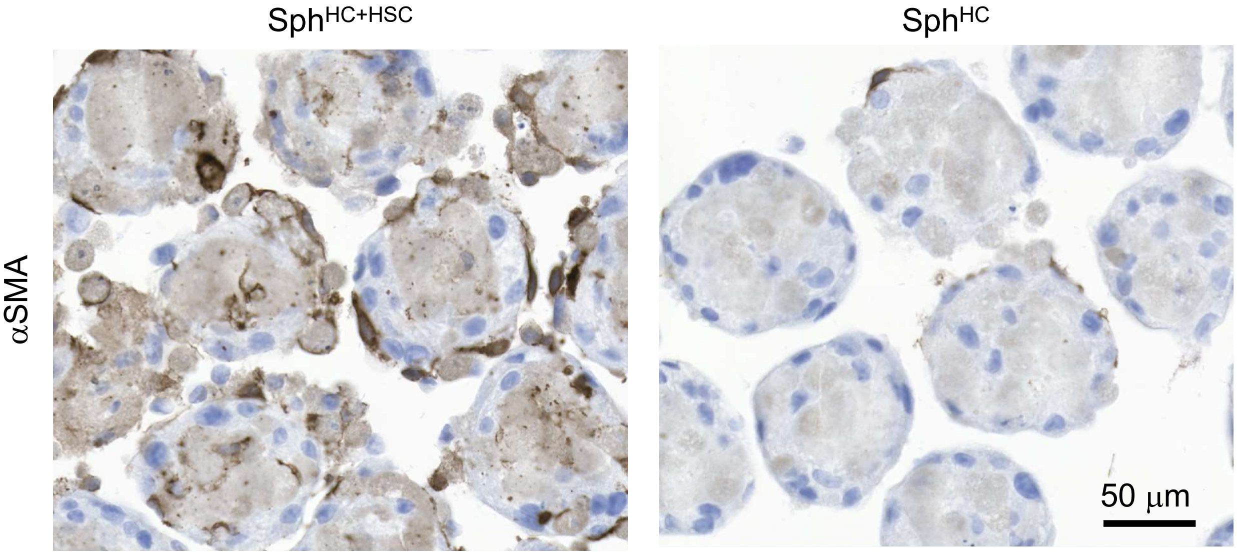

Fig. S9. Validation of the presence of activated HSCs in Sph^{HC+HSC}. Alpha-SMA IHC of Sph^{HC+HSC} and Sph^{HC}.

Disease Models & Mechanisms • Supplementary information
